# Vanity of Sawbones

**Published:** 1874-01

**Authors:** 


Vanity of Sawbones.—A doctor cannot go anywhere or put his signature to anything like an ordinary person. Many clergymen are in much the same predicament; whereas nobody but a "Rev." or an M. D. fancies that he loses dignity in society by "sinking the shop" for a moment and appearing with no professional odor about him. A social or political committee embraces W. M. Evarts, D. Webster, C. Cushing, and so on; but always G. Washington Squills, M.D., or more modestly, Dr. G. W. Squills. For example, we see in the newspapers a public notice like this:

"The undersigned beg their fellow-citizens to unite in honoring with fit ceremonies the approaching anniversary of the birthday of Washington.

| | |
|---|---|
| C. Cushing, | David D. Porter, |
| Dr. Timothy Tubbs, | W. T. Sherman, |
| Henry Ward Beecher, | T. W. H. Gallipot, *M.D.*, |
| Gideon Welles, | Charles Sumner." |

Are not such shows of titles a trifle ridiculous? Mr. Beecher does not write his "Rev." before his name, nor does Charles Sumner trail his LL.D. after it; it is only Tommy Gallipot that stickles for these things. I should think a doctor would feel cheap in thus advertising himself in an affair that has nothing to do with drugs, but is some matter of a committee upon a charity, or a flower show, or a Christmas ball, or a testimonial to the genius of a nigger minstrel, or the celebration of the Fourth of July. But no; the smallest fry of a physician displays his titular grandeur alike when signing his name to a communication on politics in the morning newspapers, or when leaving a visiting card at his friend's, in an unprofessional call.—*Galaxy for January.*

Inflammability of Ether.—In a late number of the *British Medical Journal*, Mr. Hutchinson, writing upon ether, expresses an apprehension of danger from the inflammability of the vapor, and advises against its use after dark. Ether is employed at our hospitals indifferently by night and day—as it also is in midwifery. Its practical safety is doubtless partly owing to the fact that the air, cooled by its evaporation, establishes a downward current, so that a match placed a few inches above an ether sponge at the edge of a table will not ignite it; while below, the vapor readily takes fire.

